# Fish Cell Spheroids, a Promising In Vitro Model to Mimic In Vivo Research: A Review

**DOI:** 10.3390/cells13211818

**Published:** 2024-11-04

**Authors:** Antonio Gómez-Mercader, Luis Monzón-Atienza, Daniel Montero, Jimena Bravo, Félix Acosta

**Affiliations:** Grupo de Investigación en Acuicultura (GIA), Instituto Universitario en Acuicultura Sostenible y Ecosistemas Marinos (IU-ECOAQUA), Universidad de Las Palmas de Gran Canaria (ULPGC), 35214 Telde, Spain; antonio.gomez@ulpgc.es (A.G.-M.); luis.monzon@ulpgc.es (L.M.-A.); daniel.montero@ulpgc.es (D.M.); jimena.bravo@ulpgc.es (J.B.)

**Keywords:** 3D culture, spheroids, fish cell lines, in vitro culture, fish model, bioethics

## Abstract

In vitro cell culture systems serve as instrumental platforms for probing biological phenomena and elucidating intricate cellular mechanisms. These systems afford researchers the opportunity to scrutinize cellular responses within a regulated environment, thereby circumventing the ethical and logistical challenges associated with in vivo experimentation. Three-dimensional (3D) cell cultures have emerged as a viable alternative to mimic in vivo environments. Within this context, spheroids are recognized as one of the most straightforward and efficacious models, presenting a promising substitute for conventional monolayer cultures. The application of 3D cultures of fish cells remains limited, focusing mainly on physiological and morphological characterization studies. However, given the capacity of spheroids to emulate in vivo conditions, researchers are exploring diverse applications of these 3D cultures. These include eco-toxicology, immunology, drug screening, endocrinology, and metabolism studies, employing a variety of cell types such as fibroblasts, hepatocytes, embryonic cells, gonadal cells, gastrointestinal cells, and pituitary cells. This review provides a succinct overview, concentrating on the most frequently employed methods for generating fish cell spheroids and their applications to date. The aim is to compile and highlight the significant contributions of these methods to the field and their potential for future research.

## 1. Introduction

Fish constitute the predominant group within vertebrate species, surpassing other classifications, rendering them an invaluable asset for the establishment of experimental in vitro models. These models offer a broad spectrum of applications in scientific research. Despite their potential, fish have historically received less focus compared to other animal groups due to various possible reasons. Perhaps most importantly, although fish have been bred and kept in captivity for several centuries, most of the time, this has been carried out extensively in ponds where fish grow and feed on natural prey with little or no maintenance [1]. 

In recent years, fish have been given greater consideration in animal research for a variety of reasons. Over the past 25 years, the growth in aquaculture output has surpassed most other food products, roughly tripling in live weight, and the industry has developed into a well-established global market. This rise in aquaculture production has both satisfied and driven the increasing demand for fish; consequently, this rapid growth of the industry has greatly boosted the number of studies conducted with fish. This growth in production has also increased the need for fish research in various topics related to aquaculture production, such as nutrition, immunology, animal welfare, and many others [2,3,4]. In addition, another very important reason for the increase in the use of fish in experiments is the transition from traditional biomedical models such as mice to other more novel models that are appearing as replacements or complements to previous models; for example, zebrafish is an excellent emerging model for understanding human pathology such as metabolic diseases. Due to their outstanding attributes, such as their cost-effectiveness, excellent reproducibility, rapid development, ease of use owing to the transparency of larvae, genetic similarity to humans, and possessing a large number of equivalent genes, zebrafish are proving to be incredibly valuable in studying human diseases [5,6,7].

In vitro cell culture systems serve as indispensable instruments for investigating biological phenomena and deciphering intricate cellular mechanisms [8]. These systems facilitate the examination of fish cell responses within a controlled laboratory setting, circumventing the ethical and logistical obstacles associated with in vivo experimentation. Moreover, these in vitro studies align more closely with the principles of the 3Rs (replacement, reduction, and refinement) [9] which have gained significant attention in contemporary research. These principles advocate for the minimization of animal usage in experiments, thereby promoting more humane scientific research [10]. Furthermore, advancements in the formulation of accessible cell culture media and antibiotics have facilitated the establishment of numerous continuous fish cell lines. This progress effectively circumvents many of the constraints inherent to primary cultures [8,11]. Continuous cell lines are established through the immortalization of primary cells, commonly achieved via viral transformation. This process enables these cells to undergo unlimited division in culture. These cell lines have found extensive application across diverse scientific domains, including but not limited to virology [12,13,14], toxicology [15,16,17], genetical engineering [18,19], and immunology [20,21,22]. However, currently, from more than 4000 cell lines deposited in the American Type Culture Collection (ATCC), only a few correspond to fish species [11]. Over the past decade, there has been a marked increase in efforts directed toward the development and characterization of fish cell lines. This has been further bolstered by the establishment of new institutions and facilities, such as the recently inaugurated National Repository of Fish Cell Lines (NRFC). The NRFC plays a pivotal role in promoting the extensive utilization of fish cell lines [23].

Research conducted using traditional two-dimensional (2D) cell culture systems may not accurately replicate the intricacies of in vivo environments [24,25]. These discrepancies can pose challenges when attempting to extrapolate results to real-world scenarios. In response to these limitations, three-dimensional (3D) cell culture systems have emerged as a promising alternative to their 2D counterparts [26]. Three-dimensional cell culture systems offer a more physiologically congruent environment for cell growth and maintenance. They facilitate enhanced cell-to-cell and cell-to-matrix interactions, promote improved cellular differentiation, and more accurately mimic in vivo tissue architecture. The more representative physiological model of 3D cultures leads to several advantages [27]. The morphology of 3D cultures is less sensitive to the effects of drugs compared to 2D cultures and is more effective for performing toxicological and long-term exposure studies [16,28,29,30,31]. Also, 3D cultures tend to maintain important properties of the extracellular matrix, making them closer to in vivo conditions [32,33,34]. Gen functions and genotypes are also more physiologically representative in 3D culture systems [35]. These attributes contribute to the generation of more reliable and reproducible models.

Consequently, 3D cell culture systems are increasingly being recognized as potential substitutes for whole animal usage in certain experimental contexts [36,37]. While 3D cell cultures present promising alternatives to living organisms or 2D cell cultures, their development necessitates the clear delineation of experimental objectives. This is due to the inherent complexity of these systems, which are contingent upon a multitude of parameters. These parameters encompass the source of the cells, the culture methodologies employed, the specific tissue under investigation, the decision to utilize scaffolding or not, the number of cells, and the techniques used for generating the 3D culture [38]. Within the classification of 3D cell cultures, these are divided into cultures formed on solid scaffolds (of natural or artificial origin) or scaffold-free, the latter grouping into spheroidal shapes (spheroids) or formations in spherical capsules (liquid spheres) [39].

Among the various types of 3D cultures, spheroids are distinguished as a preferred alternative to 2D cell cultures, exhibiting superior capabilities in differentiation, potency, angiogenesis, and survival [11,40,41,42]. Spheroid cultures are generated by aggregating or culturing cells in a manner that facilitates their self-assembly into a spherical configuration. This provides a more physiologically relevant model, enabling researchers to investigate cellular behavior, cell–cell interactions, and cellular responses to pharmacological agents or environmental factors.

The formation of spheroids is a consequence of interactions between membrane integrin proteins and extracellular matrix proteins. During spheroid formation, cells commence aggregation due to the presence of long-chain extracellular fibers that enable cell–surface integrins to bind. This leads to an upregulation of cadherin expression, which begins to accumulate on the cell membrane surface. Ultimately, the homophilic cadherin–cadherin binding between cells aggregates them, culminating in the formation of the spheroid [43]. 

Spheroids can be generated utilizing a variety of methodologies, including the hanging drop technique, the use of low-adherence substances, specialized culture plates, bioreactors that foster cellular aggregation, or magnetic levitation. The process of spheroid generation presents a greater challenge in comparison to generating monolayer cell cultures, thereby rendering it a complex and demanding endeavor [10,37].

One subject to take into consideration in spheroid production is the use of primary cell cultures or continuous cell lines. While primary cell cultures are directly derived from tissues, continuous cell lines come from tumors or immortalized cells that can proliferate indefinitely. Spheroids derived from primary cultures provide a more relevant physiological response since they have a high cellular heterogeneity that better mimics the in vivo environment; they also have a functional extracellular matrix that maintains tissue-specific architecture and signaling, supporting cell differentiation and function. However, such cultures have limitations such as lower growth rates and limited passages, making them challenging to maintain and work with [34,44,45]. On the other hand, continuous cell lines have an inherent ability to grow and survive for long periods of time in 3D cultures and form spheroids consistently and with less difficulty than primary cultures. Despite that, continuous cell lines have less physiological relevance compared to primary cell cultures because of many changes in their properties, such as genetic alterations, loss of specific cell markers and signaling pathways, altered extracellular matrices, and reduced cellular heterogeneity [32,33,46,47].

As a result of their properties, spheroids have been used for several different approaches in disparate areas of science. Given the ability of spheroids to imitate in vivo conditions and overcome some of the limitations of 2D cultures, one of the most explored applications is toxicology and drug screening. Currently, the prolonged impact of drugs or toxins is a major concern in many toxicological and pharmacological investigations. Spheroids are particularly advantageous for long-term studies, as they help to understand how compounds affect cells over time and reveal potential cumulative effects. While cell cultures have difficulty remaining viable for long periods of time and providing a physiologically representative response, research performed with spheroids can benefit from their ability to last for long periods of time to perform chronic exposure studies [48,49].

A large number of experiments have tested different metabolites in spheroids, demonstrating that these models correlate highly with data obtained from in vivo experiments due to the capabilities of spheroids for proliferation, metabolism, and replication, apart from many others [50,51,52,53]. Another field that has been extensively studied is the study of spheroids as tumor models, surpassing some of the limitations of traditional cultures and being able to better mimic the organization of tissue because of the hypoxic nature of the core of spheroids, which resembles conditions of in vivo tumors like distribution of oxygen and nutrients [54,55]. The use of spheroids in cancer science is useful for studying immune system triggering or signaling pathways, being especially useful for making co-cultures with other types of cells for accurate tumor models [56,57,58]. Spheroids have also been used in studies in other fields of medicine, such as regenerative medicine [59], immunology [60], parasitology [61], pathology [62], or the study of neurodegenerative diseases [63,64].

Although underexplored, fish cell spheroids are a very useful tool, as they provide a more accurate and ethical model for fish research as alternative models mimicking natural tissue architectures and allowing for more realistic simulation of in vivo conditions in fish research, which has acquired greater relevance in the last decades. Furthermore, they offer a cost-effective and scalable approach without some of the limitations of 2D cultures, allowing the study of long-term effects and more accurate responses with better replication of in vivo environments [28,29,65,66].

Currently, fish cell spheroids have been made for rainbow trout (*Oncorhynchus mykiss*) [66,67,68,69,70,71,72,73,74,75,76,77,78,79], zebrafish (*Danio rerio*) [7,10,80,81,82], clearfin livebearer (*Poeciliopsis lucida*) [30], brown trout (*Salmo truta*) [28,44], Mexican tetra (*Astyanax mexicanus*) [83], thread-sail filefish (*Stephanolepis cirrhifer*) [84], *Nothobranchius kadleci* [29], *Nothobranchius furzeri* [29], and torafugu (*Takifugu rubripes*) [85] (Table 1).

In this review, we delineate the predominant methodologies employed for the generation of fish cell spheroids. Additionally, we discuss their applications as evidenced in various studies published to date.

## 2. Conventional Spheroid Generation Methods

There are several methodologies for producing spheroids, each with its advantages and disadvantages (Table 2). In the production of spheroids, it is important to take into account several factors, such as scalability, repeatability, control of the size and shape of the spheroids, cost, and material needed... among many other factors [87,88]. Some of the most common limiting factors in the generation of spheroids are the incompatibility of spheroid production with other processes, such as the change of culture medium or characterization, as well as the requirement of specific and expensive material in most of the methods [37]. Based on tissue origin, the vast majority of studies performed with fish cell lines use the wells or microwells with shaking methodology for spheroid production, and this method has been used to generate spheroids from hepatocyte [73,82], gonad [66], gastrointestinal [69] and kidney cells [70]. The hanging drop and magnetic levitation methodologies have also been used to generate hepatocyte spheroids [10,81] and the spinner flask method for pituitary cells [85]. In this section, we describe the most common methods to generate spheroids, mainly in studies conducted with fish cell lines.

### 2.1. Hanging Drop

One of the most straightforward methodologies for spheroid generation is the hanging drop technique. This method involves the creation of cell suspension droplets on the underside of a glass or plastic surface (such as a Petri dish) or within perforated 96-well plates.

**Figure 1:**
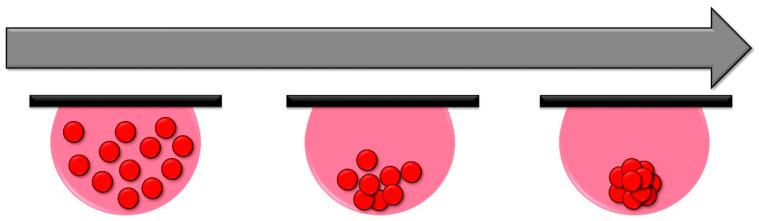
Schematic of an example of the hanging drop technique. Cells are placed in small 10 to 20 μL droplets inverted in the lid of a Petri dish with a humid environment to prevent evaporation. Then, the cells cluster at the bottom of the droplet due to the force of gravity, forming the spheroid.

To implement this technique, cell suspensions are placed upside down in volumes ranging from 10 to 20 μL onto a surface. This orientation prompts the cells to aggregate at the base of the droplet, driven by gravitational forces. As the cells migrate towards the droplet’s base, they aggregate and subsequently form a spheroid (Figure 1) [97].

The hanging drop technique is relatively straightforward and cost-effective, necessitating only fundamental laboratory apparatus such as pipettes, Petri dishes, or 96-well plates. However, due to the diminutive volume of the droplets, there exists a substantial risk of spheroid loss during transfer and relocation processes or because of evaporation [98]. Consequently, this can render the task highly challenging. To mitigate these issues, an increasing number of automated methodologies are being developed, including commercial plates designed to alleviate a significant part of these complications (Perfecta3D (3D Biomatrix, Ann Arbor, MI, USA), GravityPLUS™ (InSphero, Brunswick, GA, USA), Elplasia (Corning, NY, USA), IMAPlate™ 5RC96 (NCL New Concept Lab, Möhlin, Switzerland), etc.) [37,89,97].

### 2.2. Low-Adherence Substrates

This method involves the modification of the culture substrate to prevent cellular adhesion. Consequently, the cells aggregate amongst themselves and form spheroids due to intercellular interactions. The substrate can be either manually prepared in the laboratory or procured commercially. Currently, the most prevalent approach to producing non-adherent coated substrates involves the formation of a material layer that inhibits cellular adhesion [37]. There is a wide range of materials that can be used, including agar [66], agarose [99], or galactose [100], among many others.

**Figure 2:**
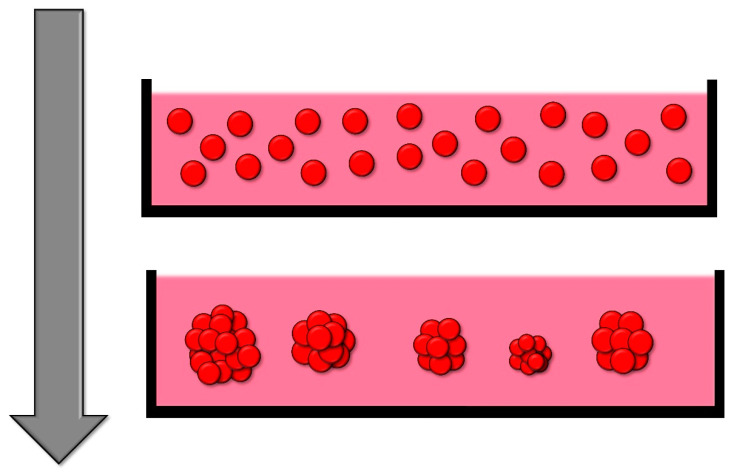
Schematic of an example of the low-adherence substrate for spheroid formation method. The low adherence substrate prevents the adhesion of cells to the surface, which promotes the formation of spheroids in the culture medium.

Non-adherent substances facilitate the production of spheroids in substantial quantities with minimal requisite manipulation (as depicted in Figure 2). However, a notable limitation of this approach is the inadequate control over the size of the spheroids unless the substrates are partitioned into diminutive regions designated for the generation of a single spheroid per region, which refers to the next method described [90].

### 2.3. Wells and Microwells in Ultra-Low Attachment Plates

**Figure 3:**
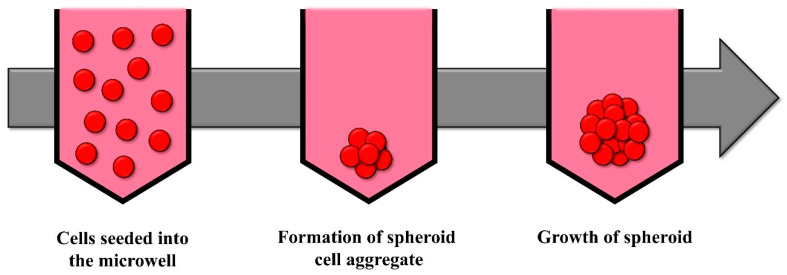
Schematic of the formation of spheroids in a well or microwell during time. Cells are incubated in the coated wells, preventing cell adhesion. Over time and usually with the aid of agitation, the cells coalesce at the bottom of the well to form a single spheroid.

Wells or microwells serve to compartmentalize cells, with dimensions on the scale of centimeters for wells and micrometers for microwells. This confinement fosters cell interaction and subsequent spheroid formation (Figure 3). To prevent cellular adhesion to the wells, the surface should be coated with a non-adherent substance, producing ultra-low attachment (ULA) plates [37,66].

Spheroid formation within wells employs cell culture plates equipped with wells that feature either a ‘U’- or ‘V’-shaped bottom. Each well contains an individual medium, necessitating those changes to the medium be conducted on a well-by-well basis. There are plates with commercial wells to produce spheroids, such as Corning, Nunclon™ Sphera™ from Thermo Fisher Scientific (Waltham, MA, USA), and CELLSTAR^®^ (Greiner Bio-One, Kremsmünster, Austria) [66].

Microwells, which can vary in size, shape, and depth, offer the ability to manipulate various properties of the spheroids, making it possible to change the culture medium during spheroid development [101]. Additionally, there are commercially available microwell plates that are specifically designed for spheroid formation, such as EZSPHERE™ (AGC, Tokyo, Japan), AggreWell™ (STEMCELL Technologies, Vancouver, BC, Canada), Elplasia plates (Corning, NY, USA), SpheroFilm (INCYTO, Hongcheondanggok-gil, Republic of Korea), and PrimeSurface^®^ 96U or 3D Petri Dish^®^ micromolds (S-bio, Constantine, MI, USA). Contrary to the wells described above, in the microwells the culture medium is usually shared and is common for all the microwells of the plate.

Commonly, rotation or shaking is used alongside this method, which facilitates the formation of spheroids. Shaking is an important parameter when forming spheroids because it promotes uniform cell distribution and enhances the nutrient and oxygen exchange of the cells, resulting in spheroids with more consistent shape and size. Rotation speed and orbit diameter are important parameters that affect and influence the distribution and homogeneity of the spheroids [91,102].

The advantage of this approach lies in its scalability, primarily facilitated by automated liquid dispensers that enable the generation of a substantial quantity of spheroids exhibiting uniformity in size and shape [103]. However, a significant challenge arises when the medium is disturbed, causing the spheroids to dislodge from the microwells and coalesce, resulting in irregular cellular aggregates. This phenomenon can introduce issues with reproducibility [37].

### 2.4. Microfluidics 

**Figure 4:**
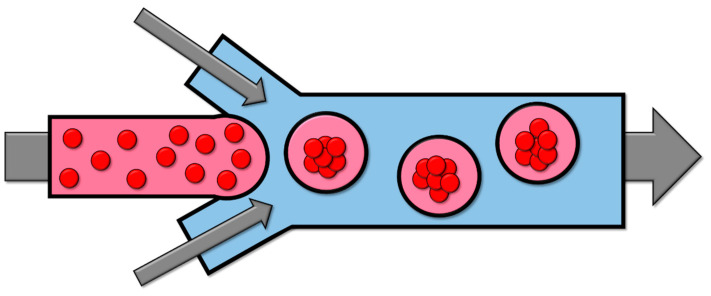
Schematic of formation of spheroids in microfluidics method. The flow channel encapsulates the cells into droplets, allowing spheroid formation inside them.

Microfluidics involves the manipulation and control of cell solutions and other fluids within flow channels that range in size from tens to hundreds of micrometers. This process generates diminutive droplets of liquid that encapsulate the cells within a medium, such as alginate or polyethylene glycol (Figure 4).

This method affords considerable control over size. However, upon encapsulation, cellular communication is impeded, cellular motility is restricted, and cell proliferation is hindered. Additionally, the encapsulating capsule forms a barrier that obstructs the exchange of gases and nutrients [92,93].

### 2.5. Magnetic Levitation

**Figure 5:**
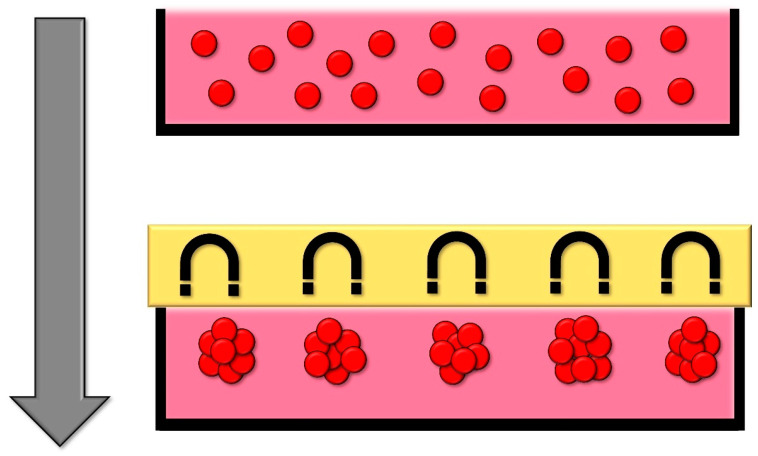
Schematic of formation of spheroids in magnetic levitation method. The cells are incubated with magnetic particles so that after adding a magnetic force, the levitation process occurs and, subsequently, the formation of the spheroids.

In this methodology, cells are endowed with magnetic properties through the incorporation of magnetic nanoparticles, a process that does not modify the inherent properties or functionalities of the cells. Subsequently, a magnetic field is employed that is positioned either at the top or base of the culture. This induces cellular aggregation through either levitation or sedimentation, contingent upon the orientation of the magnetic field [94] (Figure 5).

The advantages of this method are the ability to control the size of the spheroid, the rapid aggregation of cells, and the fact that it requires minimal handling. To carry out this methodology, there is the commercial product “Magnetic 3D Cell Culture” from Greiner Bio-One (Littleton, CO, USA) [37,39].

### 2.6. Spinner Flasks

**Figure 6:**
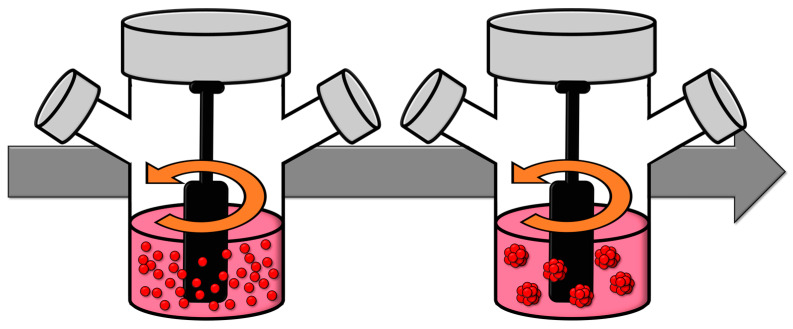
Schematic of the formation of spheroids in spinning flask method. Spinner flask rotation prevents cell adhesion to surfaces and, therefore, promotes and allows spheroid generation.

Spinner flasks facilitate the generation of spheroids via the dynamic fluid motion they produce, which induces cellular aggregation. Within these spinner flasks, the cell solution is subjected to agitation using an impeller. This agitation prevents cellular adhesion to the substrate and promotes their aggregation, culminating in the formation of a spheroid (Figure 6). By modulating parameters such as the stirring speed, volume, or cell density, one can exert control over the size and density of the resultant spheroids. However, this method is not without its drawbacks, the primary one being the mechanical damage inflicted upon cells due to collisions and the relatively short lifespan of the cultures [95].

An alternative variant of this method, termed the microgravity reactor, involves the rotation of the culture medium about a horizontal axis. This rotation induces a flow that maintains the cells in a state of ‘constant tumble’, subjecting them to minimal hydrodynamic forces and simulating microgravity conditions. It is postulated that this environment stimulates the expression of genes conducive to spheroid formation. Microgravity reactors facilitate the large-scale production of spheroids while minimizing collisions between spheroids and offering good control over their size. However, a notable disadvantage of this methodology is its substantial financial cost [37,96].

## 3. Fish Cell Spheroid Applications

Spheroids are useful 3D cell cultures capable of emulating in vivo conditions, opening the door to a wide range of opportunities as alternative methods. Although they have been widely used in mammals, spheroids still remain seldom used in other animal groups of great importance in current science, such as fish. Due to their novel nature, much of the research conducted with spheroids deals with their standardization and morphological and physiological characterization; however, there are already studies dealing with different areas of science, such as toxicology, endocrinology, and pathology (Table 3) [65,66,70,76,85,86].

### 3.1. Characterization and Optimization of Spheroid Culture

As a relatively nascent technique and an under-explored model, a significant proportion of studies pertaining to spheroid culture are focused on the establishment of standardized and replicable culture conditions. Furthermore, there exists a multitude of fish cell lines that have yet to be explored within the context of 3D cultures. 

The expression of Cyp1a was later analyzed by Lammel et al. [72] in a continuous rainbow trout liver cell line (RTL-W1), showing a higher expression in spheroids compared to traditional cultures. 

Spheroids have demonstrated high viability and suitability as models for studying hepatic biotransformation, bioaccumulation, and chronic toxicity. They represent a potent and different methodology for spheroid generation that was evaluated using ZFL and ZEM2S zebrafish spheroids to determine the most optimal protocol. It was observed that the hanging drop and orbital shaking (OS) methods, when implemented in a 96-well plate without coating, were not viable for spheroid formation. Conversely, the hanging drop and orbital shaking methods, when used with ultra-low adherent (ULA) plates, were successful in producing spheroids. Both the hanging drop combined with OS in ULA plates and OS in ULA plates methodologies proved to be reproducible, reliable, and trustworthy for spheroid formation, exhibiting minimal variability in size and circularity [10].

In 2023, Součková et al. [29] established several innovative cell lines for *Nothobranchius kadleci* and *Nothobranchius furzeri* and conducted tests to generate viable 3D spheroids. These spheroids were produced and subsequently evaluated with cytotoxic and growth-inhibitory compounds. The results demonstrated the efficacy of these cultures for applications in ecotoxicology, among numerous other scientific disciplines. 

### 3.2. Fish Pathology

Given that spheroids represent a relatively novel and as yet under-explored model, there is currently not so much information regarding their application in fish pathology assays. However, their potential to effectively mimic in vivo responses during an infection suggests that they could serve as highly useful models for such studies [13]. This underscores the need for further research in this area to fully elucidate the capabilities and potential applications of these spheroid models in the field of aquatic pathology; nevertheless, some studies have been conducted.

In an in vitro study conducted by Faber et al. [66], spheroids derived from the RTG-2 cell line of rainbow trout were subjected to infection testing. These spheroids were exposed to spores of *Saprolegnia parasitica* to evaluate the viability of the infection. Eight hours post-exposure, mycelia were detected within the spheroids, indicating successful attachment and penetration into the 3D cultures. By the 15-h mark, the mycelia had fully infiltrated the spheroids, a finding that was corroborated by means of immunofluorescent staining. These results suggest that RTG-2 spheroids could serve as an alternative method for elucidating the mechanisms of *Saprolegnia parasitica* infection. However, further research and characterization of this model are required to fully comprehend its potential applications and to establish it as a reliable model for such studies.

Suryakodi et al. [70] conducted an in vitro study in which infections with the SJNNV virus were performed on spheroids derived from the RTK kidney cell line of rainbow trout. The outcomes were then compared with those obtained from conventional 2D culture infections. The propagation of the SJNNV virus was detected earlier in the spheroids than in the 2D cultures, as evidenced by means of immunofluorescence microscopy. Furthermore, the results from quantitative reverse transcription polymerase chain reaction (qRT-PCR) indicated higher viral loads in the spheroids. These findings suggest that spheroids could potentially be suitable for large-scale production of whole-virus vaccines, thereby contributing to advancements in vaccine development strategies. 

### 3.3. Metabolism

Baron et al. [78] developed a spheroid model with rainbow trout hepatocytes, obtaining mature spheroids at 6 days, which were stable during a period of 40 days following data obtained for biochemical markers. They were also able to convert galactose to glucose, showing similar levels of glucose secretion as in the organotypic liver slice models. Albumin synthesis, which is essential for liver-specific function, was higher than that in monolayer cultures. Regarding LDH leakage, when low levels were found, this meant that membrane integrity was maintained; meanwhile, high levels of LDH suggested inhibition of enzyme release. Assessing the results, trout spheroids were shown to be reliable replacements for fish in toxicological studies.

To develop an in vitro method that closely mirrors the in vivo micro-environment, spheroids derived from the ZFL liver cell line of zebrafish were employed to investigate the metabolism of the pharmaceutical compound carbamazepine. The activity of the enzyme CYP1a1 was observed to be higher in the spheroids compared to traditional cultures. Additionally, the concentration of the metabolite carbamazepine-10,11-epoxide was found to be more significantly upregulated in the spheroids. These findings suggest that these spheroids could potentially serve as a useful model for simulating the in vivo micro-environment, thereby providing valuable insights into the metabolic processes of pharmaceutical compounds in aquatic organisms [81].

Spheroids are typically categorized into three distinct micro-environments based on their oxygen metabolism: quiescent, hypoxic, and anoxic. In a study conducted by Langan et al. [69], the influence of spheroid size on metabolism, specifically the oxygen microenvironment, was examined using the RTgutGC cell line derived from the intestine of rainbow trout. The metabolic pathways of pharmaceutical compounds were investigated using high-performance liquid chromatography as a function of spheroid size. The findings indicated that the oxygen-viable zone within the spheroid diminished over time, while conversely, the hypoxic necrotic zone expanded with increasing spheroid size. The study concluded that smaller spheroids (with a diameter of less than 200 μm) should be employed for metabolic studies to minimize the impact of these micro-environments.

In a comprehensive study conducted by Alves et al. [44], the metabolic activity of fish hepatocyte spheroids was examined using a resazurin assay. The findings indicated that the spheroids remained metabolically active throughout the duration of the experiment; for days 12 to 20, the viability and functionality of the spheroids stabilized, thereby demonstrating the functionality of the mitochondria and the integrity of the cellular membranes. Metabolism and detoxification, efflux transport, and estrogenic signaling results showed that even further research is needed; however, the model developed was promising for toxicological xenobiotic research in fish. 

### 3.4. Toxicology 

**Figure 7:**
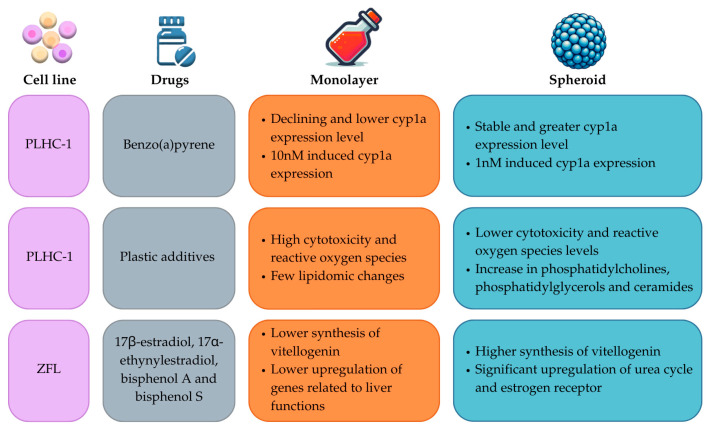
Examples of comparison of results in toxicology experiments with both fish monolayer and spheroid cell cultures [30,31,82].

The establishment and validation of dependable in vitro methodologies, which serve as alternatives to traditional approaches in hepatotoxicity studies, represents one of the most extensively investigated applications of spheroids derived from fish cell lines. Consequently, a majority of the spheroid-related studies involving fish cell lines have been conducted using fish hepatocytes [65]. The outcomes of these studies predominantly indicate that spheroids more accurately emulate in vivo conditions compared to conventional cultures (Figure 7).

In the study conducted by Flouriot et al. [79], it was demonstrated that in rainbow trout (*Oncorhynchus mykiss*), the induction of estrogen receptor and vitellogenin messenger RNAs (mRNAs) by 17β-estradiol mirrored the levels observed in hepatocytes in vivo. Notably, these levels exhibited stability and functionality throughout the experimental duration, thereby outperforming traditional cell cultures in terms of response. This evidence suggests that the experimental model employed in this study could potentially offer a more accurate simulation of physiological responses in vivo, thereby providing significant implications for future research in the field of endocrine disruption. This could further aid in the development of more efficacious strategies for managing endocrine-disrupting chemicals in aquatic ecosystems. In a subsequent study conducted by Flouriot et al. [77], spheroid cultures with a maturation period of 30 days were utilized. The researchers concluded that these cultures represented promising models for in vitro studies due to their resemblance to in vivo biotransformation pathways and their regulation by both endogenous and exogenous compounds. This finding underscores the potential of these spheroid cultures as effective tools for studying physiological processes and responses, thereby enhancing our understanding of the biological systems involved. The metabolism of the spheroids was further studied by Cravedi et al. [74].

The metabolism of the hepatocyte spheroids of rainbow trout was further analyzed by Uchea et al. [75,76]. An in-depth analysis of the metabolism of hepatocyte spheroids derived from rainbow trout was undertaken. The findings highlighted the remarkable preservation of activity and longevity within these cultures. Furthermore, these spheroid cultures exhibited a heightened expression of genes implicated in xenobiotic metabolism when compared to conventional 2D cultures. As a result, these spheroid cultures were proposed as a viable alternative model for studies pertaining to the bioaccumulation of environmental contaminants and their metabolites. This suggests that these cultures could provide more accurate insights into the impact of environmental pollutants on aquatic life, thereby informing more effective strategies for environmental conservation and pollution control.

In clearfin livebearer, hepatocytes were utilized to construct a 3D model for the purpose of analyzing adaptive and toxic responses to various hepatic toxicants over a specified time period. The findings indicate that these 3D models exhibited an elevated basal expression of the xenobiotic metabolizing enzyme, cytochrome P450 1A (Cyp1a), in comparison to traditional 2D cultures. Upon exposure to 1 nM benzo(a)pyrene, Cyp1a expression was induced in the spheroids, whereas a concentration of 10 nM was required to induce expression in 2D cultures. A comparative analysis of the two culture types revealed distinct variations in adaptive response, microtissue architecture, and cell death. Consequently, these 3D clearfin liver cell cultures may serve as a suitable alternative to more costly and time-consuming in vivo experiments, offering enhanced sensitivity and capabilities compared to traditional culture methods [31]. 

In a comprehensive study conducted by Baron et al. [73], the metabolic pathways of seven distinct pharmaceuticals were scrutinized utilizing a substrate depletion assay for the following pharmaceuticals: atenolol, carbamazepine, diazepam, diclofenac, metoprolol, phenylbutazone, and propranolol. The data were procured from substrate depletion kinetics, which facilitated the calculation of intrinsic hepatic clearance; the obtained results compared with the trout liver microsomal fraction (S9) indicated that fish liver spheroids could potentially serve as a relevant and metabolically efficient in vitro system for evaluating the transformation of pharmaceuticals in fish. This suggests that these spheroids could provide valuable insights into the metabolic processes of pharmaceuticals in aquatic organisms, thereby contributing to the development of more effective strategies for managing pharmaceutical pollution in aquatic environments.

In 2019, Hultman et al. [67] determined that spheroids derived from hepatocytes of rainbow trout exhibited high metabolic activity over extended periods of exposure, effectively biotransforming pyrene into its respective metabolites. The results of this study were juxtaposed with those of other referenced studies, thereby facilitating a comparative analysis with other in vitro models. The findings suggest that these spheroids may serve as more suitable models for mimicking in vivo conditions, thereby providing a more accurate representation of physiological responses and processes. This underscores the potential of these spheroids as effective tools for studying the impact of environmental pollutants on aquatic life and informing more effective strategies for environmental conservation and pollution control. 

Spheroids derived from zebrafish hepatocytes were evaluated for their potential to assess the toxic effects of endocrine-disrupting chemicals in fish. The spheroids were constructed utilizing a scaffold composed of collagen-polyethylene glycol (PEG) and using two different matrices with different softness. The spheroids were exposed to 17β-estradiol to analyze the parameters of morphology, urea production, and intracellular oxidative stress. Even though the results obtained seem promising to improve the testing of endocrine-disrupting chemicals in fish, additional research is required to ascertain the suitability of these zebrafish hepatocyte spheroids for application in ecotoxicology studies [80].

A comprehensive whole-transcriptomic-sequence analysis was conducted on ZFL spheroids to investigate the transcriptional regulation of genes implicated in hepatic functions and toxicological responses. A toxicological assay was performed using 17β-estradiol, 17α-ethynylestradiol, bisphenol A, and bisphenol S to evaluate the impact of these compounds in comparison to monolayer cultures. Post-exposure, the spheroids exhibited a significant upregulation of the genes associated with the hepatic functions, thereby suggesting their superior suitability for simulating in vivo scenarios. This underscores the potential of these spheroids as effective tools for studying the impact of environmental pollutants on aquatic life, thereby informing more effective strategies for environmental conservation and pollution control [82].

Pereira et al. [28] utilized hepatocyte spheroids derived from brown trout to develop models for mechanistic studies pertaining to physiology and toxicology. These spheroids were exposed to 5α-dihydrotestosterone. The results indicated an upregulation of *Fabp1* and *Acsl1* and a downregulation of *Acox1-3I*, *PPARγ*, and sphericity. These findings suggest that the spheroids generated in this study were able to effectively mimic the in vivo conditions of brown trout hepatocytes. This underscores the potential of these spheroids as effective tools for studying physiological and toxicological responses in aquatic life, thereby informing more effective strategies for environmental conservation and pollution control. 

Järvinen et al. [71] utilized spheroid cultures derived from the rainbow trout liver cell line RTH-149, and cryopreserved primary rainbow trout hepatocytes (RTHEP) were evaluated as models for assessing the cytotoxicity of various human pharmaceuticals, with the aim of evaluating their hepatotoxicity. The findings indicate that the screening process was beneficial in providing preliminary data regarding the impact of the tested substances. However, the direct extrapolation of data from human studies proved to be ineffective at the time of the experiment. This underscores the complexity of translating findings from human studies to aquatic models and highlights the need for further research in this area. 

In an effort to assess the toxicity of a composite mixture of plastic additives, a continuous cell line derived from *Poeciliopsis lucida* (PLHC-1) was employed as an in vitro model designed to mimic in vivo hepatic cellular function. Throughout the course of the experiment, the spheroids demonstrated high viability and metabolic activity. Subsequent lipidomic analysis revealed that the spheroids’ lipidomic response was markedly sensitive, surpassing that of 2D cultures, and bore a striking resemblance to in vivo results obtained from liver samples. Also, the toxicity of the plastic additives was tested in spheroids cultured with and without FBS, showing that FBS had a protective role in the lipidomic profile of the cells. Spheroids exposed to plastic additives in the absence of FBS had several changes in their lipidomic profile, with upregulation and downregulation of a large number of lipids; meanwhile, spheroids cultured with FBS showed only a significant decrease in diglycerides and ether-linked phosphatidylethanolamines. Based on these findings, it was concluded that the PLHC-1 spheroids serve as robust models for emulating in vivo results, thereby providing valuable insights into the impact of plastic additives on aquatic life [30,86].

### 3.5. Endocrinology

Fish pituitary spheroids were made by Yamaguchi et al. [85] to develop an in vitro protocol that could be extrapolated to in vivo surveillance of the annual reproductive physiological conditions of torafugu, which rely on its neuroendocrine system. The results showed that the production of luteinizing hormone (LH) reaches its highest level during the last phase of maturation according to the highest serum steroid levels. Additionally, the study demonstrated that in 3D cell propagation assays using female serum, total pituitary cells exhibited peak proliferation at the onset of puberty. These results prove useful for the study of reproductive physiological states and can be applied to investigate the effects of various compounds that might impact the effectiveness of reproduction in aquaculture.

## 4. Future Prospects

Although they are still an understudied model, spheroids have been increasingly recognized in many studies for their ability to mimic in vivo conditions more accurately than traditional 2D cell cultures. This has significant implications for the study of fish physiology, toxicology, and disease and could potentially transform the way we approach fish aquaculture.

One of the key advantages of using fish spheroids is their ability to emulate the complex microenvironments found in living organisms. This includes the replication of oxygen gradients, cell–cell interactions, and cell–matrix interactions, which are often lost in 2D cultures. By preserving these features, spheroids can provide a more accurate representation of physiological responses, thereby enhancing our understanding of fish biology and informing more effective strategies for different topics like fish farming. In the realm of toxicology, spheroids have shown great promise as models for assessing the impact of environmental pollutants on fish health. For instance, spheroids derived from fish liver cells have been used to study the metabolism of various pharmaceutical compounds, revealing insights into the impact of these substances on fish health. This could potentially inform the development of more effective strategies for managing pharmaceutical pollution in aquatic environments, thereby protecting the health of farmed fish and ensuring the sustainability of fish farming operations.

Spheroids also hold potential for the study of fish diseases. For example, spheroids derived from fish pituitary cells have been used to study the mechanisms of various infections, providing valuable insights into the pathogenesis of these diseases and informing the development of more effective treatments. This could potentially enhance the health and productivity of farmed fish, thereby contributing to the economic viability of fish farming operations. The use of spheroids in fish aquaculture is a burgeoning field that holds immense potential for revolutionizing our understanding of fish biology and improving the sustainability of fish farming practices. The use of spheroids in fish aquaculture also has significant implications for the 3Rs of animal experimentation: Replacement, Reduction, and Refinement. By providing a more accurate in vitro model that closely mimics in vivo conditions, spheroids can potentially reduce the need for live animal experiments, thereby aligning with the principle of replacement. Furthermore, by providing more accurate and reliable data, spheroids can potentially reduce the number of animals needed for experiments, thereby aligning with the principle of reduction. Finally, by providing a less invasive and more humane alternative to live animal experiments, spheroids can potentially enhance the welfare of experimental animals, thereby aligning with the principle of refinement. Despite these promising developments, the use of spheroids in fish aquaculture is still in its early stages, and much research remains to be carried out. For instance, further research is needed to standardize the protocols for spheroid production and to fully elucidate the capabilities and potential applications of these models. Additionally, more studies are needed to validate the findings obtained from spheroid models and to ensure their reproducibility.

In conclusion, the use of fish spheroids represents a promising avenue for future research. By providing a more accurate representation of in vivo conditions, spheroids have the potential to enhance our understanding of fish biology, inform more effective strategies for fish farming, and contribute to the sustainability of aquaculture. As research in this area continues to advance, it is anticipated that spheroids will play an increasingly important role in the field of fish research and in aligning aquaculture practices with the principles of the 3Rs in animal experimentation.

## Figures and Tables

**Table 1:** List of fish cell lines used in experiments with 3D spheroid cultures.

Cell Line	Species	Type of Cell	Method of Generation	Utility	Reference
Primary culture	*Astyanax mexicanus*	Hepatocyte	Wells + Shaking	Characterization Metabolism Transcriptomics	[83]
ZFL	*Danio rerio*	Hepatocyte	Scaffold-based	Toxicology	[80]
ZFL	*Danio rerio*	Hepatocyte	Magnetic levitation	Toxicology	[81]
ZFL	*Danio rerio*	Hepatocyte	Hanging drop + shaking Wells + Shaking	Characterization	[10]
ZFL	*Danio rerio*	Hepatocyte	Wells + Shaking	Characterization Transcriptomics	[82]
ZFL ZEM2S	*Danio rerio*	Hepatocyte Embryo	Wells + Shaking	Characterization	[7]
14A-NFUD3, 19A-NFUD3, 15II-NFUD2, 17II-NFUD2	*Nothobranchius furzeri*	Embryonic	Micromolded agarose gels	Characterization	[29]
14-NKAD3, 1-NKAD3, 7A-NKAD3	*Nothobranchius kadleci*	Embryonic	Micromolded agarose gels	Characterization	[29]
Primary culture	*Oncorhynchus mykiss*	Hepatocyte	Wells + Shaking	Toxicology	[67]
RTL-W1	*Oncorhynchus mykiss*	Hepatocyte	Petri dish + Shaking	Toxicology	[72]
Primary culture	*Oncorhynchus mykiss*	Hepatocyte	Wells + Shaking	Metabolism	[73]
Primary culture	*Oncorhynchus mykiss*	Hepatocyte	Wells + Shaking	Metabolism	[74]
Primary culture	*Oncorhynchus mykiss*	Hepatocyte	Petri dish + Shaking	Metabolism	[75]
Primary culture	*Oncorhynchus mykiss*	Hepatocyte	Petri dish + Shaking	Metabolism	[76]
Primary culture	*Oncorhynchus mykiss*	Hepatocyte	Petri dish + Shaking	Metabolism	[77]
Primary culture	*Oncorhynchus mykiss*	Hepatocyte	Wells + Shaking	Characterization	[78]
Primary culture	*Oncorhynchus mykiss*	Hepatocyte	Petri dish + Shaking	Characterization	[79]
RTG-2	*Oncorhynchus mykiss*	Gonad	Wells + Shaking	Characterization Immunology	[66]
RTG-2	*Oncorhynchus mykiss*	Gonad	Wells + Shaking	Characterization Metabolism	[68]
RTgutGC	*Oncorhynchus mykiss*	Gastro-intestinal	Wells + Shaking	Characterization Metabolism	[69]
RTK	*Oncorhynchus mykiss*	Kidney	Wells + Shaking	Characterization Immunology	[70]
RTHEP	*Oncorhynchus mykiss*	Liver	Wells	Toxicology	[71]
PLHC-1	*Poeciliopsis lucida*	Hepatocyte	Wells	Toxicology	[31]
PLHC-1	*Poeciliopsis lucida*	Hepatocyte	Wells	Toxicology	[30]
PLHC-1	*Poeciliopsis lucida*	Hepatocyte	Wells	Toxicology	[86]
Primary culture	*Salmo trutta*	Hepatocyte	Wells + Shaking	Characterization	[44]
Primary culture	*Salmo trutta*	Hepatocyte	Wells + Shaking	Characterization Metabolism	[28]
deSc	*Stephanolepis cirrhifer*	Fibroblast	Flask	Characterization	[84]
Primary culture	*Takifugu rubripes*	Pituitary	Spinner flask and spheroid dish	Endocrinology	[85]

**Table 2:** List of spheroid generation methods with advantages and disadvantages.

Method	Advantages	Disadvantages	References
Hanging drop	Cost effective Simple material required Control of spheroid size	Low Throughput Limited scalability Risk of evaporation	[37,88,89]
Low-adherence substrates	Cost effective Simple material required High throughput	Low control in the size of the spheroid Limited scalability Time-consuming	[37,90]
Wells	Good control over size of the spheroid High throughput	Limited scalability Time-consuming	[37,66,91]
Microfluidics	Good control over size of the spheroid High cell viability	Low Throughput Specific material needed Isolation of spheroids encapsulated	[92,93]
Magnetic levitation	Good control over size of the spheroid Rapid aggregation of cells Minimal handling	Limited scalability Specific material needed	[37,39,94]
Spinner flask	Good scalability High cell viability	Low control in the size of the spheroid Specific material needed	[95,96]

**Table 3:** Summary of studies with different applications conducted to date in fish spheroids.

Cell Line	Drug	Results	Reference
Primary brown trout hepatocyte culture	Estradiol (10^−6^ M)	mRNAs of trout estrogen receptor and vitellogenin stable over time with similar levels to in vivo. Continued and efficient production and release of vitellogenin throughout the entire culture period.	[79]
Primary brown trout hepatocyte culture	β-naphthoflavone (0.36 μM)	7-Ethoxyresorufin O-deethylase (EROD) activity not inducted in 3-day spheroids, whilst significant increase was seen in 28-day 3D cultures.	[77]
Primary brown trout hepatocyte culture	β-naphthoflavone (0.36 μM) Testosterone (0.17 μM)	Cytochrome P450 levels lowered until day 5; since then, remained stable one month. EROD increased significantly for one month, resembling data obtained from fresh hepatocytes. Testosterone hydroxylase activity was 30% of the activity found in fresh brown trout hepatocytes.	[74]
Primary brown trout hepatocyte culture	7-ethoxyresorufin (8 µM) 7-ethoxycoumarin (100 µM) Ibuprofen sodium salt (10 µM)	Higher EROD levels than in hepatocyte cultures but lower than freshly isolated hepatocytes. Spheroids should increase predictability rather than S9 fraction because of their prolonged metabolic activity.	[75]
Primary brown trout hepatocyte culture	Fluorescent compounds and specific inhibitors	Positive functionality of efflux transporters in mature spheroids.	[76]
PLHC-1	Benzo(a)pyrene (1 nM)	Cell death and decrease in Cyp1a expression.	[31]
Primary brown trout hepatocyte culture	Atenolol, carbamazepine, diazepam, diclofenac, metoprolol, phenylbutazone, and propranolol (100 μg∙L^−1^)	Diclofenac intrinsic hepatic clearance was similar to the S9 fraction, while propranolol was 5-fold higher. Atenolol, metoprolol, diazepam, and carbamazepine not metabolized by spheroids.	[73]
Primary brown trout hepatocyte culture	Pyrene (25 nM)	The optimal number of spheroids per reaction was 100 and duration was 30 h. Effective biotransformation of pyrene into OH-PYR-Glu metabolite by spheroids from 2 h to 30 h of exposure. More prolonged duration of exposure than fraction S9 is needed, but also, the data resemble in vivo conditions more.	[67]
ZFL	17β-estradiol, 17α-ethynylestradiol, bisphenol A, and bisphenol S (0.0002, 0.002 and 2 mM)	Significant upregulation of several genes in spheroids compared to 2D cultures such as ctnnb1, urea cycle, hepatic cytochrome P450, glycogen and glucose metabolism, nuclear receptors, and transcriptional factors. Increase in vitellogenesis over time.	[82]
Primary brown trout hepatocyte culture	5α-dihydrotestosterone (DHT) (10 and 100 µM)	Spheroids treated with the highest DHT concentration (100 µM) decreased sphericity and loss of 3D dense disposal; also, PPARγ and Acox1-3I genes were downregulated. For both treatments, there was an increase in the immunochemistry signal for caspase-3, upregulation of the Acsl1 gene. The treatment with lower dose upregulated Fabp1 gene.	[28]
RTH-149	Carbamazepine, propranolol, clozapine, fluoxetine, haloperidol, levomepromazine, quetiapine, sertraline, venlafaxine, clotrimazole, ketoconazole, diclofenac, ibuprofen, naproxen (9 concentrations ranging from 0.78 to 1000 μM)	Sensitivity of spheroids to sertraline, fluoxetine, levomepromazine, quetiapine, and diclofenac, especially the first three with cell viabilities (EC_50_s) ≤ 10 µM. The spheroids became less susceptible with the time of culture except for fluoxetine, which was affected significantly more compared to the spheroids at 72 h post-culture than 24 h.	[71]
Primary rainbow trout hepatocyte culture	Carbamazepine, propranolol, clozapine, fluoxetine, haloperidol, levomepromazine, quetiapine, sertraline, venlafaxine, clotrimazole, ketoconazole, diclofenac, ibuprofen, naproxen (9 concentrations ranging from 0.78 to 1000 μM)	Lower toxicity of compounds than in RTH-149 spheroids, with clozapine and haloperidol not having toxic effects. All the compounds had higher EC_50_s compared with RTH-149 cell line, except for ketoconazole.	[71]
PLHC-1	di(2-ethylhexyl) phthalate (DEHP), dibutyl phthalate (DBP), bisphenol A (BPA), bisphenol F (BPF), 4-tert-octylphenol (OP), 4-tert-nonylphenol (NP), bisphenol A bis(3-chloro-2-hydroxypropyl) ether (BADGE 2HCl), triclosan (TCS), 3,3,5,5’-tetrabromobisphenol A (TBBPA), tritolyl phosphate (TPP) (Concentrations ranging from 1 to 50 µM)	Plastic mixture showed a decrease in EC_50_ in the spheroids, but not as much as in monolayer culture. A smaller amount of induced ROS species was found in spheroids compared with monolayer cultures. Enrichment of ceramides and upregulation of 19 lipids and downregulation of 6 lipids in spheroids lipidomic response. Increase in phosphatidylcholines, phosphatidylethanolamines, and ceramides, and decrease in cholesteryl esters. Increase in phosphatidylcholines/phosphatidylethanolamines ratios.	[30]
PLHC-1	bis(2-ethylhexyl) phthalate (DEHP), dibutyl phthalate (DBP), bisphenol A (BPA), bisphenol F (BPF), 4-tert-octylphenol (OP), 4-tert-nonylphenol (NP), bisphenol A bis(3-chloro-2-hydroxypropyl) ether (BADGE·2HCl), triclosan (TCS), 3,3′,5,5′-tetrabromobisphenol A (TBBPA), and tritolyl phosphate (TPP) (Concentrations ranging from 1 to 50 µM)	Spheroids cultured in absence of fetal bovine serum (FBS) had an upregulation of phosphatidylcholines, alkyl, and alkenyl ether-linked ceramides and a downregulation of ether-linked phosphatidylethanolamines and phosphatidylglycerols; meanwhile, spheroids with included FBS in culture media showed only a decrease in diglycerides and ether-linked phosphatidylethanolamines, which shows a greater effect of the plastic additives to the spheroids with no FBS in culture media. Also, plastic additives had a higher cytotoxicity in cells without FBS.	[86]
**Cell line**	**Pathogen**	**Results**	**Reference**
RTG-2	*Saprolegnia parasitica*	Positive viability of spheroids. Successful infiltration of mycelia into the spheroids.	[66]
RTK-1	Stripped jack Nervous Necrosis Virus (SJNNV)	Visible viral infection at 2 days post-infection (dpi) in spheroids; 60% of spheroid cells tested positive for SJNNV and expressed viral intracellular protein. Higher production of SJNNV in spheroids rather than in 2D culture.	[70]
**Cell line**	**Hormone**	**Results**	**Reference**
Torafugu primary pituitary spheroids	Torafugu serum	Luteinizing hormone synthesis was found to be dose-dependent with torafugu serum, while follicle-stimulating hormone levels did not correlate with serum exposure. During puberty onset the proliferation of the pituitary spheroids was at its maxim.	[85]
**Cell line**	**Drug**	**Results**	**Reference**
ZFL	Carbamazepine	High CYP1a1 activity in spheroids, superior to monolayer culture. Upregulation of metabolite carbamazepine-10,11-epoxide in spheroids.	[81]
RTgutGC	Pharmaceutical compounds	Oxygen-viable zone within the spheroid reduced over time and hypoxic necrotic zone expanded with greater sizes. Spheroids with diameters less than 200 μm suggested for metabolic studies.	[69]
Hepatocyte primary culture	Resazurin	Stable metabolic activity of the spheroids, especially between days 12 and 20. Metabolism and detoxification, efflux transport, and estrogenic signaling results were viable for toxicological xenobiotic research.	[44]

## Data Availability

Not applicable.
